# Draft Genome Sequences of Four Bacterial Strains Isolated from a Polymicrobial Culture of Naked (N-Type) *Emiliania huxleyi* CCMP1516

**DOI:** 10.1128/genomeA.00674-16

**Published:** 2016-07-14

**Authors:** Fabini D. Orata, Albert Remus R. Rosana, Yue Xu, Danielle N. Simkus, Anna R. Bramucci, Yan Boucher, Rebecca J. Case

**Affiliations:** Department of Biological Sciences, University of Alberta, Edmonton, Alberta, Canada

## Abstract

Strains of *Sulfitobacter* spp., *Erythrobacter* sp., and *Marinobacter* sp. were isolated from a polymicrobial culture of the naked (N-type) haptophyte *Emiliania huxleyi* strain CCMP1516. The genomes encode genes for the production of phytohormones, vitamins, and the consumption of their hosts’ metabolic by-products, suggesting symbiotic interactions within this polymicrobial culture.

## GENOME ANNOUNCEMENT

The haptophyte *Emiliania huxleyi* is a coccolithophore, a group of marine microalgae that has the ability to make calcite disks (coccoliths). Although it is possible to cultivate members of this species axenically, many strains lose their ability to produce coccoliths through prolonged culturing, commonly referred to as “laboratory domestication” (1). The *E. huxleyi* CCMP1516 strain culture and the isogenic line M217 constitute a model system for the study of biomineralization, and both contain bacteria. CCMP1516 was maintained by the Bigelow National Center for Marine Algae and Microbiota and eventually lost its ability to calcify (2). Bacteria associated with this algal strain were cultivated to describe its microbiota, and genomes of four bacterial strains were sequenced to identify metabolic and signaling interactions with their host.

DNA was extracted from single-colony isolates using the DNeasy Blood and Tissue Kit (QIAGEN, Hilden, Germany) according to the manufacturer’s protocol. Sequencing libraries from the genomic DNA extracts were prepared using the Nextera XT DNA Library Preparation Kit (Illumina, San Diego, CA, USA). Whole-genome sequencing was performed using the NextSeq 500/550 High Output Kit version 2 (for 300 cycles) and NextSeq sequencing technology (Illumina), generating 150-bp paired-end reads. *De novo* assembly of the reads into contiguous sequences (contigs) was done using the CLC Genomics Workbench version 7.5.2 (CLC bio, Aarhus, Denmark). The draft genomes were then annotated using RAST version 2.0 (3) or PGAP (http://www.ncbi.nlm.nih.gov/genome/annotation_prok). All of the genomes sequenced exceeded 100× coverage, and the characteristics of the assemblies obtained are described in Table 1. Species identities were determined by an average nucleotide identity >95% using JSpecies version 1.2.1 (4), and genus identities were determined by an average amino acid identity (AAI) >60% using the AAI calculator (http://enve-omics.ce.gatech.edu/aai) (5) with previously sequenced genomes in the GenBank database. This analysis identified one gammaproteobacterium from the Alteromonadales order (*Marinobacter* sp. EhN04), one alphaproteobacterium from the Sphingomonadales order (*Erythrobacter* sp. EhN03), and two *Sulfitobacter* spp. (*Sulfitobacter geojensis* EhN01 and *Sulfitobacter pontiacus* EhN02).

**TABLE 1:** Genome features and GenBank accession numbers of the four strains isolated from a polymicrobial culture of N-type *Emiliania huxleyi* CCMP1516

Isolate	Accession no.	Genome size (kb)	No. of contigs	*N*_50_ (kb)	G+C (mol%)
*Sulfitobacter geojensis* EhN01	LXYM00000000	4,266	46	196	57.9
*Sulfitobacter pontiacus* EhN02	LXYK00000000	3,466	44	157	60.5
*Erythrobacter* sp. EhN03	LXYL00000000	3,007	15	465	63.7
*Marinobacter* sp. EhN04	LXYN00000000	4,619	38	540	57.2

The *Sulfitobacter* isolates encode genes to degrade lignin, a likely component of the *E. huxleyi* cell wall (6, 7). All bacteria except *Erythrobacter* sp. EhN03 can metabolize dimethylsulfoniopropionate produced by *E. huxleyi*. All isolated bacteria also have the ability to transport siderophores, but only one of them, *Marinobacter* sp. EhN04, has the ability to synthesize siderophores. Like many other algal symbionts, all of these bacteria encode a pathway to produce multiple vitamin Bs and the phytohormone auxin (8–10). The two *Sulfitobacter* isolates also harbor type IV secretion systems. These characteristics suggest that these four bacteria engage in symbiotic interactions with their hosts, involving the production and consumption of a number of primary and secondary metabolites.

### Nucleotide sequence accession numbers.

The whole-genome shotgun projects have been deposited in DDBJ/ENA/GenBank under the accession numbers listed in Table 1.
